# Real-Time Vehicle Positioning and Mapping Using Graph Optimization

**DOI:** 10.3390/s21082815

**Published:** 2021-04-16

**Authors:** Anweshan Das, Jos Elfring, Gijs Dubbelman

**Affiliations:** 1 Signal Processing Systems Group, Department of Electrical Engineering, University of Eindhoven, 5600 MB Eindhoven, The Netherlands; g.dubbelman@tue.nl; 2 Control Systems Technology Group, Department of Mechanical Engineering, University of Eindhoven, 5600 MB Eindhoven, The Netherlands; j.elfring@tue.nl; 3 Product Unit Autonomous Driving, TomTom, 1011 AC Amsterdam, The Netherlands

**Keywords:** multi-sensor fusion, pose-graph optimization, vehicle localization

## Abstract

In this work, we propose and evaluate a pose-graph optimization-based real-time multi-sensor fusion framework for vehicle positioning using low-cost automotive-grade sensors. Pose-graphs can model multiple absolute and relative vehicle positioning sensor measurements and can be optimized using nonlinear techniques. We model pose-graphs using measurements from a precise stereo camera-based visual odometry system, a robust odometry system using the in-vehicle velocity and yaw-rate sensor, and an automotive-grade GNSS receiver. Our evaluation is based on a dataset with 180 km of vehicle trajectories recorded in highway, urban, and rural areas, accompanied by postprocessed Real-Time Kinematic GNSS as ground truth. We compare the architecture’s performance with (i) vehicle odometry and GNSS fusion and (ii) stereo visual odometry, vehicle odometry, and GNSS fusion; for offline and real-time optimization strategies. The results exhibit a 20.86% reduction in the localization error’s standard deviation and a significant reduction in outliers when compared with automotive-grade GNSS receivers.

## 1. Introduction

Autonomous driving technologies are evolving rapidly with the ultimate goal of developing a safe and reliable, fully autonomous vehicle, i.e., SAE level 4 and eventually level 5 [1]. A real-time, accurate, and robust positioning system is the backbone of a fully autonomous vehicle and many Advanced Driver Assistance Systems (ADAS). It is the basis for environment perception, path planning, and autonomous decision making. Global Navigation Satellite System (GNSS) is most widely used for vehicle positioning (GPS, GLONASS, Galileo, BeiDou are all examples of GNSS systems). However, these systems are not always reliable as they are dependent on satellite visibility. Obstruction of GNSS signals because of trees and large buildings, or GNSS signals that get reflected before being received by the GNSS receiver (multipath error), severely degrades the receiver’s performance [2,3]. Researchers have fused inertial measurement unit (IMU) sensor data with GNSS data to increase its precision and reliability [4,5]. Positioning systems that fuse IMU and GNSS data are referred to as the Inertial Navigation System (INS). The state-of-the-art INS uses tactical-grade IMUs with Real-time kinematics (RTK) GNSS receivers to estimate positions accurately. Their data are often postprocessed to achieve centimeter-level accuracy. However, these systems are too expensive to be deployed in consumer-grade vehicles. This article focuses on improving the availability, accuracy, and reliability of the vehicle positioning system fusing low-cost automotive-grade sensor data.

The most popular approaches to sensor fusion for the purpose of vehicle positioning are filter-based or graph-based. In filter-based approaches, typically, different Bayesian filter variants take a recursive approach and usually adopt the Markov assumption. In other words, all measurements are summarized by a state that is refined each time a new measurement arrives. The measurements are then discarded after being processed. This approach limits computational costs and is particularly popular in real-time settings [6,7,8,9,10,11]. Graph-based methods, typically pose-graphs, formulate a nonlinear least-squares optimization problem using a set of measurements rather than processing them one-by-one [12,13]. Pose-graph consists of nodes that represent the state (position and orientation or pose), and two nodes are always connected by an edge representing the measurement between the two. In the optimization process, the state estimates are optimized by considering a set of measurements rather than relying on a Markov assumption. This approach has been demonstrated to be more accurate in various problems such as Simultaneous Localization and Mapping or SLAM [14,15,16,17] and bundle adjustment [18,19]. Filter-based solutions have become much less popular ever since. For a comparison of filter-based methods and graph-based methods in the context of visual SLAM, we refer to [20].

One major drawback of pose-graph optimization is that it is computationally demanding. At best, the optimization time increases linearly with the size of the pose-graph. Real-time or online execution is possible if the number of nodes and measurements is limited. To tackle this problem, researchers have proposed sliding-window pose-graph optimization strategies, which limit the size of the pose-graph by considering a subset of measurements to make optimization computationally tractable [21,22,23]. Global optimization requires optimizing over all nodes in the graph. Sliding window pose-graph optimization techniques optimize the sequence of vehicle poses over the n most recent nodes only and therefore perform local optimization instead.

The benefit of absolute GNSS positioning, compared to integrating relative positioning (vehicle odometry and visual odometry), is that the position errors are bounded, whereas integrating relative positioning will accumulate errors indefinitely (drift). Vehicle odometry is typically estimated using Micro-electro-mechanical systems (MEMS) gyroscopes, IMUs, and wheel encoders. Low-cost MEMS IMUs are not accurate and do not exhibit long-term accuracy, i.e., its accuracy significantly decreases over distances in the order of 1 km. Wheel encoders are robust, but accuracy degrades with wheel slip and changing tire pressure. Visual odometry estimates the camera/vehicle’s pose by tracking static visual features between consecutive image frames taken from single or multiple camera systems [24,25,26,27]. These systems have proven to be accurate in low traffic urban scenarios where there are many static image features to track. It often fails in dark environments, real-world dense traffic scenarios, and highways, where there are considerably fewer static features to track. Relative position estimates do not contain direct information on the absolute position. Instead, they act as soft constraints between absolute poses. If the relative pose estimates would contain no errors (i.e., act as hard constraints) and if GNSS readings would not be correlated in time, then absolute position errors could be reduced with the order of $\sigma /\sqrt{n}$, with σ being the standard deviation of the GNSS error and *n* the number of GNSS readings. In reality, this model is highly naive because: (1) the relative pose estimates do contain errors and should only be used as soft constraints and (2) the GNSS readings are highly correlated in time [28], reducing the statistical information of a single GNSS reading when fused with other positioning sources.

**Figure 1:**
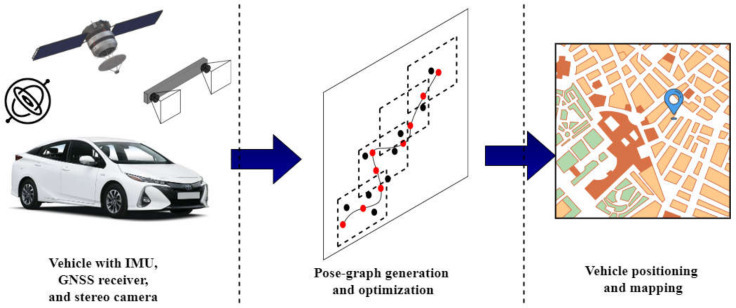
Multi-sensor fusion for vehicle positioning and mapping using automotive-grade sensors. The sensor data are used to model a pose-graph, which is then optimized in real-time to estimate the vehicle’s accurate pose and generate a map.

In this article, we propose and evaluate a pose-graph optimization-based vehicle positioning and mapping framework using automotive-grade GNSS receiver, stereo camera, and in-vehicle yaw-rate and velocity sensor data, depicted in Figure 1. In contrast to SLAM, where loop closure detection is used to compensate for the drift and generate an accurate map, we fuse absolute GNSS measurements with relative odometry measurements. The GNSS measurements act as loop closures, which reduces dependency on image-based landmark and feature detection algorithms. We combine and extend our previous work [29,30] on pose-graph based vehicle localization. In [29], we propose and evaluate multiple off-line pose-graph modeling strategies to fuse vehicle odometry and GNSS data for robust vehicle positioning. In [30], we extended our work in [29] and proposed a real-time pose-graph generation and optimization framework, "Incremental Hopping Window pose-graph Optimization" for vehicle positioning. This article extends the framework to model stereo visual odometry, vehicle odometry, and GNSS measurements into a pose-graph which is then optimized using the incremental hopping window pose-graph fusion strategy [30]. We also extensively compare the framework’s performance with (a) vehicle odometry and GNSS fusion (b) stereo visual odometry, vehicle odometry, and GNSS fusion for real-time vehicle positioning on large datasets covering more than 180 km in different scenarios such as urban-canyons and highways. An overview of the framework is depicted in Figure 4.

The rest of the article is structured as follows. In Section 2, we describe some of the related works. A brief explanation about pose-graph optimization and pose-graph structure is provided in Section 3 and Section 4, respectively. The pose-graph generation and real-time optimization process are described in Section 5 and Section 6, respectively. The experiments and results are provided in Section 7 and the conclusion in Section 8.

## 2. Related Work

A lot of research has been done to enhance vehicle positioning capabilities in urban scenarios using a GNSS receiver. Hieu et al. [31] present a loosely coupled model for INS/GPS integration using an extended Kalman filter. They show that accurate positioning and navigation results are possible from 9 to 14 s of GPS outages with the position errors spread from 3 to 10 m (Root mean square). Andrew Howard [25] proposes a stereo visual odometry algorithm for estimating frame-to-frame camera motion from successive stereo image pairs using a dense stereo matching algorithm. This approach generalized and simplified the approach described by Hirschmüller [32], which uses feature matching rather than tracking and employs stereo range data for inlier detection, by introducing a complete inlier detection scheme (based on the notion of cliques) and simplifying the point-to-point inlier test to permit faster comparisons. Agarwal et al. [33] proposed a real-time, low-cost system mobile robot localization system for outdoor environments. Their system relied on a stereo vision to robustly estimate frame-to-frame motion in real-time. The motion estimation problem from a stereo camera (visual odometry) is formulated in the disparity space and used inertial measurements to fill in motion estimates when visual odometry failed. The motion is then fused with a low-cost GPS sensor using a Kalman filter. This system was mainly designed low speed outdoor mobile robotics applications.

Rehder et al. [34] present a pose-graph optimization-based approach to estimate the global pose of a vehicle using stereo-visual odometry which is affected by bias due to lack of close-range features and very infrequent GPS measurements. They show that the graph-based state estimation framework is capable of inferring global orientation using a unified representation of local and global measurements and recovers from inaccurate initial estimates of the state. Chiu et al. [35] tackled the real-time pose-graph optimization problem by combining a long-term smoother and a short-term smoother using the Sliding-Window Factor Graphs in iSAM2 (Kaess et al. [36]). Indelman et al. [37] use the incremental smoothing technique from [36] to fuse multiple odometry and pose sources. They choose a similar graph representation as proposed in this contribution, with the difference that they keep the full graph in memory over the entire trajectory, making the approach more memory consuming. Cucci and Matteucci [38] propose the graph-based ROAMFREE framework for multi-sensor pose tracking and sensor calibration. They keep the size of the graph bounded by simply discarding older nodes and edges, thus potentially obtaining overconfident estimates. Merfels et al. [21] propose a pose-graph optimization-based multi-sensor fusion approach that combines measurements from multiple localization systems in a plug-and-play manner. They formulate the problem as a sliding window pose-graph optimization, enabling efficient optimization and providing accurate pose estimates with high availability. They use a novel marginalization approach that marginalizes information in the last optimization window into a single prior node before generating a new window. In this article, our goal is to develop a graph-based real-time vehicle positioning and mapping framework. We research different pose-graph modeling approaches that model relative and absolute sensor measurements into a pose-graph. We propose incremental hopping window pose-graph optimization strategy for real-time vehicle positioning and perform extensive evaluations on a dataset covering 180 km of vehicle trajectories. Our proposed approach is conceptually similar to [21] but differs in the following aspect: (a) we model GNSS measurements considering their uncertainty, whereas they provide constant weight to all GNSS measurements, (b) we define the size of the optimization window with respect to the distance travelled, whereas they define it in time, (c) they marginalize the information of the last optimization window into a single prior node before optimizing a new window, whereas we use a section of the last optimization window as prior.

## 3. Pose Graph

**Figure 2:**
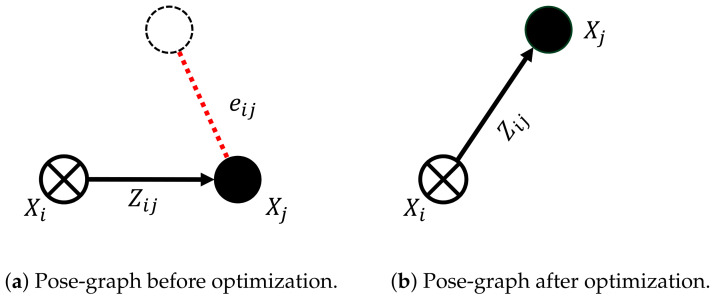
A simple graph before (**a**) and after (**b**) optimization [29]. The initial node *X_i_* is kept fixed. The node *X_j_* is at its initial position before optimization. The nodes *X_i_* and *X_j_* are connected by an edge (measurement) *Z_ij_*. The black dashed circle visualizes the measured position of node *X_j_* contained in the edge *Z_ij_*. The error vector *e_ij_* before optimization is depicted as a red dashed line. After the optimization, this error is minimized by moving node *X_j_* to the position according to the measurement contained in *Z_ij_*.

In this section and Section 4, we provide a small introduction to pose-graph optimization and pose-graph structures before explaining the proposed framework in Section 5 and Section 6. The sensor fusion algorithm optimizes a pose-graph that models the vehicle’s motion from vehicle odometry, visual odometry, and GNSS receiver readings using the least-squares optimization technique to estimate the vehicle’s pose in real-time. The pose-graph consists of nodes, denoted by *X*, which model the absolute vehicle pose by elements of SE(2), i.e., Euclidean motions in 2-D, and of edges denoted by *Z*, which model the relative poses between nodes, also with elements of SE(2). We have chosen to use SE(2) instead of SE(3) because the automotive-grade low-cost GNSS receiver can only provide reliable 2D position estimates, i.e., latitude and longitude. The altitude estimates from these receivers are unreliable. Each measurement *Z* is accompanied with uncertainty expressed in the tangent space of SE(2) using an information matrix denoted with Ω. The edges always connect two nodes, i.e., the edge $Z_{ij}$ denotes a relative pose that moves the node $X_{i}$ onto $X_{j}$. The error $e_{ij}$ between the poses of the nodes $X_{i}$ and $X_{j}$ with respect to the measured relative pose $Z_{ij}$ is computed with:

(1) $$e_{ij}=\log\left(Z_{ij}^{-1}\left(X_{i}^{-1}X_{j}\right)\right)\;,$$

where log() denotes the logarithmic map from SE(2) to its tangent space, i.e., $e_{ij}$ is a three-dimensional vector consisting of the angular and positional difference between $X_{i}$ and $X_{j}$, as shown in Figure 2. The goal of graph optimization is to minimize the following nonlinear objective function:

(2) $$X^{*}=\underset{X}{\mathrm{argmin}}\sum_{\langle i,j\rangle \in C}e_{ij}^{T}\Omega_{ij}e_{ij}\;,$$

where *C* represents the set of all index pairs for which measurements are available. This optimization task can be performed with the usual nonlinear solvers like Levenberg–Marqaurdt, Gauss–Newton, or Dogleg [39]. In our work, we use the Gauss–Newton solver contained in the g2o graph optimization framework developed by Kuemmerle et al. [12].

In order to explain the graph structure, we use a graphical notation, which is introduced next. Nodes or absolute poses in the graph are visualized using solid circles. Whenever a node is kept fixed, i.e., its pose is not optimized for, the circle contains a cross. The edges or relative poses are visualized using arrows. In order to better visualize the actual measurement contained in an edge and its error w.r.t. the absolute nodes, we visualize the measurement as a dashed circle and the error as a red dashed line. Figure 2 shows a simple graph before and after optimization. In this example, the error is minimized by taking node $X_{j}$ from its initial position to the position corresponding to the measurement contained in the edge $Z_{ij}$.

## 4. Pose Graph Structure

**Figure 3:**
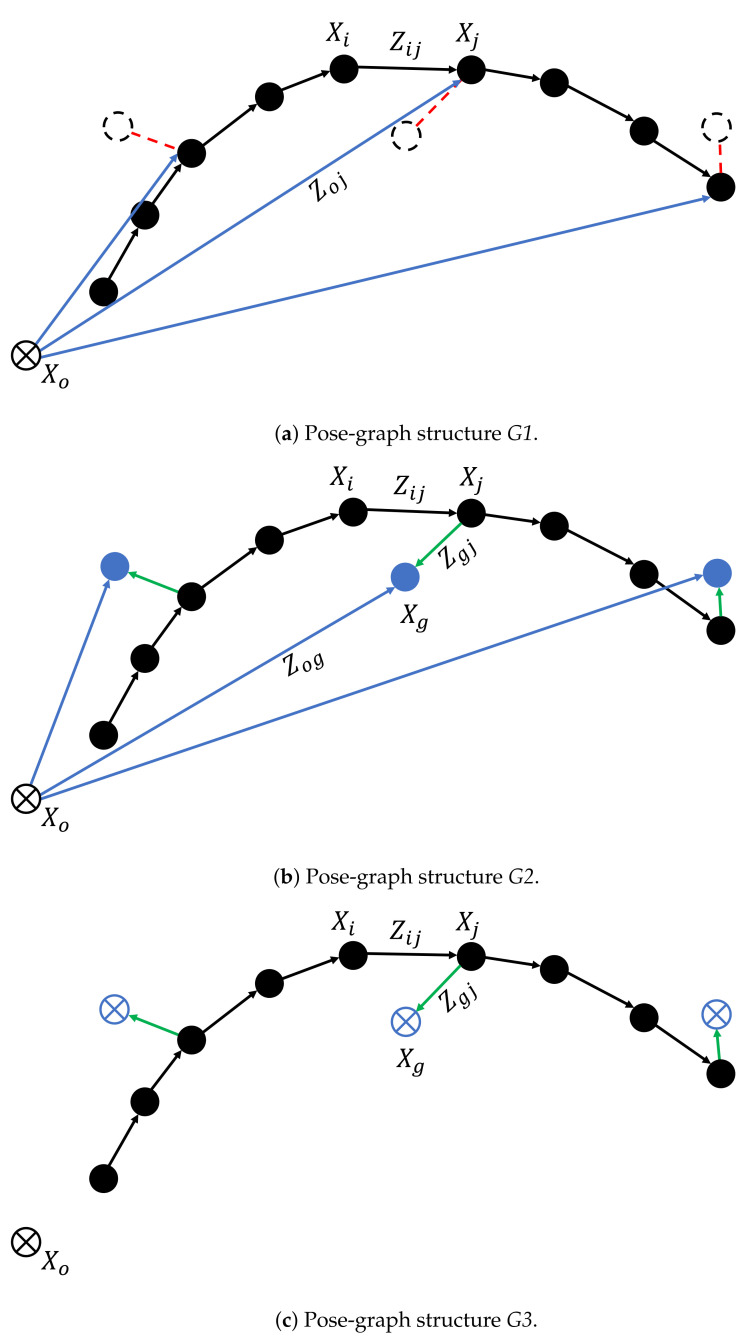
PGraph modeling strategies *G1*, *G2*, and *G3*, in (**a**–**c**), respectively [29]. The black circles are the absolute vehicle poses initialized from the odometry, and the black arrows are the corresponding edges. The blue arrows are the GNSS edges connecting the UTM origin node (black circle with a cross) with the corresponding nodes. In (**a**), the black dashed circles represent the GNSS readings, and the error is depicted by a red dashed line. In (**b**), the blue circles are the GNSS readings nodes. The green arrows represent the (virtual) identity edges. In (**c**), The blue circles with crosses are the GNSS nodes that are kept fixed during optimization.

We explore three different strategies to model the optimizable pose-graph: G1, G2, and G3, as shown in Figure 3. The three approaches differ in the manner in which the GNSS readings are modeled. We note that all the three approaches are intrinsically the same, i.e., the global minima of their objective functions are located at the same point in the parameter space. However, due to modeling the GNSS readings differently, their convergence characteristics can differ, and in our experimental evaluation, the aim is to research which structure exhibits favorable convergence characteristics.

### 4.1. Modeling Approach G1

In the first approach, both vehicle odometry and GNSS readings are modeled as measurements (edges), see Figure 3a. The absolute poses of the vehicle are computed from the odometry. Hence they initially exactly coincide with the measured relative poses. The goal of the graph optimization is then to minimize the errors related to the GNSS readings. This will alter the relative poses between nodes and hence introduce error for odometry measurements, but this error is compensated for by the reduction in the error related to the GNSS readings. After convergence, the graph is in an optimal balance between the errors related to the relative vehicle odometry and the errors related to the absolute GNSS readings.

### 4.2. Modeling Approach G2

In the second approach, GNSS readings are modeled as nodes, and their absolute positions are optimized for, see Figure 3b. In order to link the poses of the vehicle, initially provided by the vehicle odometry, to the GNSS readings, we introduce an extra edge between the GNSS nodes and their corresponding vehicle poses. These are depicted by the green arrows in Figure 3b. These edges model (virtual) identity measurements, stating that the particular GNSS poses and the vehicle pose are the same: they act as very strong soft-constraints. The potential benefit of this approach is that there is more flexibility in the graph as there are more measurements to optimize for. It can improve convergence when the vehicle poses suffer from poor initial guess. However, by modeling the GNSS readings as nodes, we increase the number of nodes that are optimized for (proportional to the number of GNSS readings) and therefore increase the computational load.

### 4.3. Modeling Approach G3

In the third approach depicted in Figure 3c, the GNSS readings are also modeled as nodes, but now they are kept fixed during optimization. The uncertainties of the GNSS readings are now transferred to the (virtual) identity edges, which no longer act as strong soft-constraints but as regular edges. *G2* and *G3* have the same number of nodes; however, for *G3*, all GNSS nodes are fixed, which will result in lower computational costs. Compared to approach *G1*, this offers an alternative way of modeling the GNSS readings without optimizing for their position as in approach *G2*.

## 5. Pose Graph Generation

**Figure 4:**
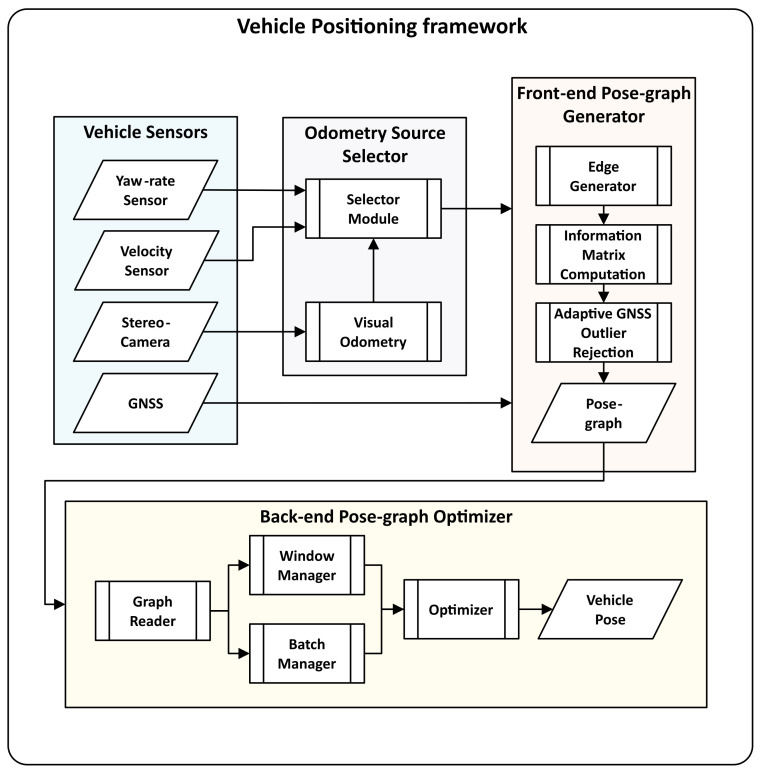
Pose-graph based sensor fusion framework for vehicle positioning and mapping.

The vehicle positioning and mapping framework, depicted in Figure 4, realizes the in-vehicle multi-sensor fusion process. The framework is composed of four blocks: vehicle sensors, odometry source selector, front-end pose-graph generator, and back-end pose-graph optimizer. The odometry source selector block estimates the motion of the vehicle using a sequence of stereo-camera images, which is validated using the vehicle’s velocity and yaw-rate sensor. If the difference in motion computed from the two sources is more than a threshold, the block switches to the velocity and yaw-rate sensor from the stereo-camera. The selected odometry source and GNSS measurements are used to model a pose-graph in the front-end Pose-graph Generator block, and the pose-graph is optimized in real-time in the back-end Pose-graph Optimizer block. The GNSS receiver is used to estimate the vehicle’s pose in the Universal Transverse Mercator (UTM) coordinate system. The process of modeling the vehicle sensor data into a pose-graph is explained in this section.

### 5.1. Visual Odometry

**Figure 5:**
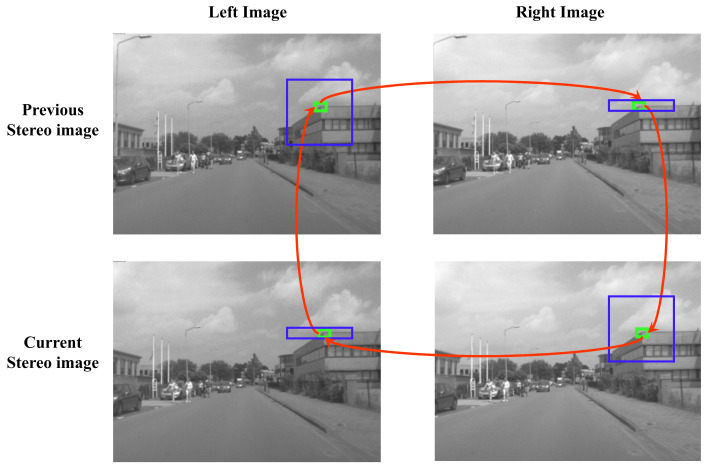
Circle feature matching process. The green boxes represent the matched feature point and the blue boxes represent the search space. The red arrows represent the sequence of the matching process.

The process of estimating the relative poses or motion of a camera using a sequence of images is called visual odometry. In this article, we use a front-facing stereo camera set up to estimate the motion of the vehicle, commonly referred to as stereo-visual odometry (SVO). A modified multi-threaded version of the *libviso2* library by Geiger et al. [40] is used for SVO. The motion of the camera is estimated by detecting and matching static image feature points between the two consecutive stereo-image pairs (previous and the current stereo images). The feature points are detected using a corner and a blob detector and are then matched between the four images of the two stereo-image pairs using a Sobel filter response [40]. Figure 5 shows the “circle” feature matching strategy. First, for all feature point candidates in the current left stereo-image, we find the best match in the previous left stereo-image within a *M* × *M* search window. The points are then matched with the previous right stereo-image, the current right stereo-image, and the current left stereo-image again. When matching candidates between the left and right images, the epipolar constraint of an error tolerance of 1 pixel is used. The candidate is considered a valid match if the last feature coincides with the first feature. The valid candidates in the previous stereo frame are reprojected onto the current stereo frame. The camera motion is then estimated by minimizing the sum of reprojection errors. An outlier rejection scheme based on random sample consensus (RANSAC) is applied before the final motion optimization step. The obtained motion estimates are then refined using a Kalman filter [40].

### 5.2. Odometry Source Selector

The stereo-visual odometry estimates the motion of the camera/vehicle by matching static feature points in consecutive frames. In ideal scenarios, it drifts less and is more accurate than odometry derived using automotive-grade IMUs and odometers. However, in heavy traffic scenarios, when the camera’s field of view (FOV) is occluded by large moving objects like trucks or large vehicles, its performance degrades. If most of the feature points that are matched are on a moving truck, it estimates the motion of the vehicle relative to the truck. Applying RANSAC is not enough to estimate the exact motion from such biased data. We use the in-vehicle velocity and yaw-rate sensor data to validate the motion estimates from the SVO to tackle this issue. Here we assume that the velocity and the yaw-rate sensor have a small number of outliers compared to SVO. The transformation estimate between the two consecutive stereo frames is compared with the transformation estimated by integrating the velocity and yaw-rate measurements for the period. If SVO and vehicle odometry is similar, SVO is assumed to be more accurate and is therefore preferred; otherwise, the velocity and yaw-rate sensor measurements are used.

### 5.3. Edge Generator

The inputs to the front-end pose-graph generator block are velocity and yaw-rate measurements of the vehicle and its GNSS receiver measurements. It models these measurements into nodes and edges of a pose-graph. The pseudocode of the pose-graph generation process for the graph modeling approach *G2* is given in Algorithm 1. The odometry nodes are generated using the yaw-rate and velocity measurements from the Odometry source selector block. We preintegrate the yaw-rate and velocity measurements [41] to compute the change in heading and the distance traveled by the vehicle. The nodes containing the vehicle’s pose are generated when there is a change in heading of more than 5 deg ($\Delta a_{thr}$), or the vehicle has traveled at-least 1.5 m ($\Delta d_{thr}$) or when a GNSS measurement is received. The edge between the two consecutive odometry nodes is estimated by computing the transformation matrix between the two poses. The GNSS nodes are generated for each GNSS fix from the GNSS receiver, operating at 1Hz. The GNSS edge represents the measurement between the origin node (UTM tile origin) and the GNSS receiver antenna. The identity measurement or edge is generated between the GNSS node and the corresponding odometry node.

### 5.4. Information Matrix Determination

The GNSS receiver estimates the expected accuracy of its fix for latitude, longitude, and altitude at the 95% confidence bound. These expected accuracies are provided in meters and denoted with epx, epy, epv. These uncertainty values are computed from GNSS reading Dilution of Precision (DOP). The information matrix values for the UTM-X and UTM-Y coordinate measurements for each GNSS edge is computed as ${\left(epx/2\right)}^{-2}$ and ${\left(epy/2\right)}^{-2}$, respectively, where we assume that there is no correlation in both directions.

The information matrix for all odometry edges is computed as the inverse of the covariance matrix of each odometry measurement. The covariance matrix is derived from the average 1.1% drift of the total distance traveled. We assume that when the velocity is zero, the vehicle cannot move or rotate from its position. In this case, we give the odometry a high certainty ($1e^{5}$ for the corresponding elements in the information matrix) for the corresponding edges. This prevents the vehicle from having abnormal movements after pose-graph optimization. For example, the vehicle will not show any lateral displacement with zero longitudinal displacements.

### 5.5. Adaptive GNSS Outlier Rejection

So far, we have assumed that the GNSS receiver provides a good estimate for its uncertainty using the epx, epy, epv values. However, this holds true when there is sufficient satellite visibility. The accuracy of GNSS receiver is severely degraded when trees and buildings block the line-of-sight to satellites or multi-path error is induced due to signal reflection. The epx, epy, epv values do not necessarily reflect this. Ignoring this will severely degrade the performance of the fusion framework. We call these erroneous GNSS measurements with overconfident epx, epy, epv values *outliers*, and we propose an approach to detect and ignore them before fusing. This approach is based on the fact that GNSS readings have low short-term accuracy, but the yaw-rate and velocity sensors in the vehicle have very high short-term accuracy. Thus the vehicle odometry can be used as an observer to detect GNSS outliers. We do this by computing relative measurements from the absolute GNSS readings for each second and compute the difference with respect to the relative measurements of the vehicle odometry for the same time span. If the error of change in heading and displacement is below 1.5 deg ($\Delta gh_{thr}$) and 3 m ($\Delta gt_{thr}$), respectively, the GNSS reading is incorporated in the pose-graph. We have tuned these thresholds for high precision, i.e., we try to make sure that all outliers are rejected at the expense of also rejecting good some measurements.

**Algorithm 1:** Pose-graph generation

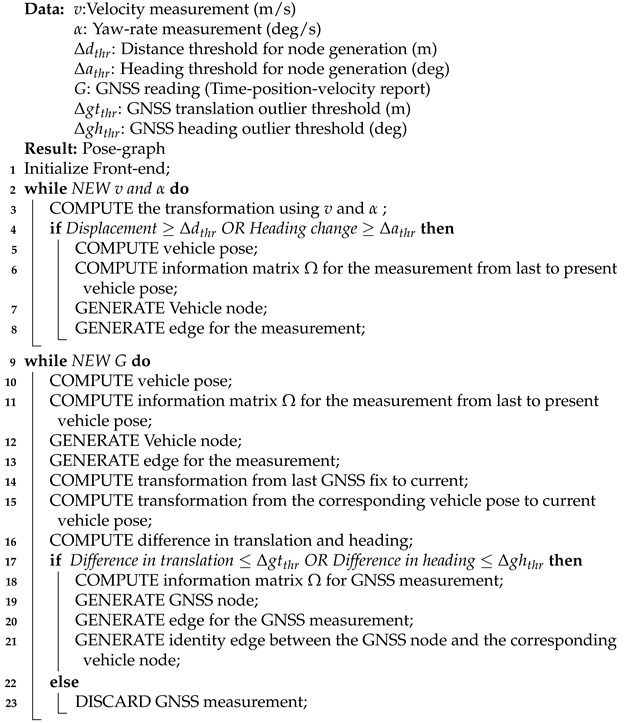



## 6. Pose Graph Optimization

The pose-graph generated in the front-end pose-graph generator is optimized in the back-end pose-graph optimizer to estimate the accurate pose of the vehicle. Typically the most accurate results are obtained when all sensor measurements from the beginning to the end of a session are modeled into a pose-graph and then optimized. However, it cannot be used for real-time vehicle positioning, as the size of the pose-graph increases indefinitely with time. At best, the optimization time and memory requirement increases linearly with the size of the pose-graph with the use of sparse solvers [12]. This increase in size also increases the computational cost and, in turn, prevents meeting real-time system constraints. To achieve real-time performance, pose-graphs are often optimized using sliding-window pose-graph optimization strategies. Merfels et al. [21] marginalize the information of the last pose-graph window into a prior of the initial node for the next pose-graph window. Similar results can be achieved by using a portion of the optimized pose graph from the last window to generate a new pose-graph window, as is done in our approach. The overall goal of window-based optimization is to best as possible approximate global optimization and to obtain close-to similar results. In this section, we describe our local optimization approach, incremental hopping window pose-graph optimization, in detail. The pseudocode is given in Algorithm 2.

### 6.1. Window Manager

**Figure 6:**
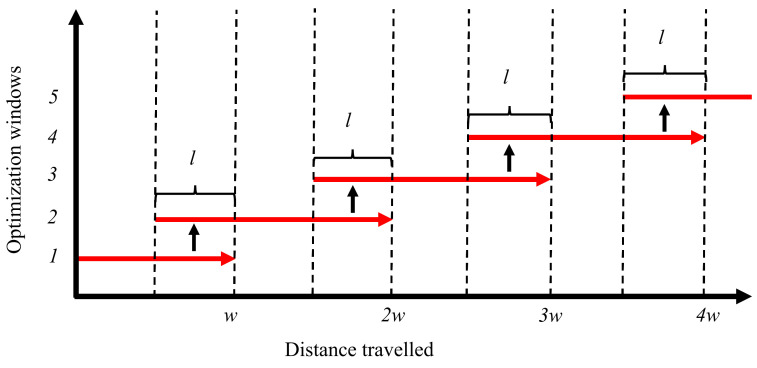
The red arrows represent a window; a new window is generated at every multiple of *w* meters traveled by the vehicle, with all the nodes contained in the last *l* meters of the last optimized window, which creates a hopping effect [30].

The main purpose of this block is to maintain the size of the optimization window. It limits the size of the optimization window to achieve real-time performance. It also maintains the list of the optimized vertices that are used from the last optimized window to generate the new one. Figure 6 provides an explanation of how pose-graph optimization windows are created. Let *w* meters be the distance traveled for which a new window is generated, and *l* meters be the length of the last optimized pose-graph that is kept to generate the new window. When the distance traveled by the vehicle in the present window is greater than *w* (only considering new measurements), it copies the nodes of the pose graph from the last *l* meters of the optimized window to a new optimization window. All new odometry and GNSS nodes and their edges are added to this new window. The first node of a window is always kept fixed, i.e., it is not optimized for.

### 6.2. Batch Manager

**Figure 7:**
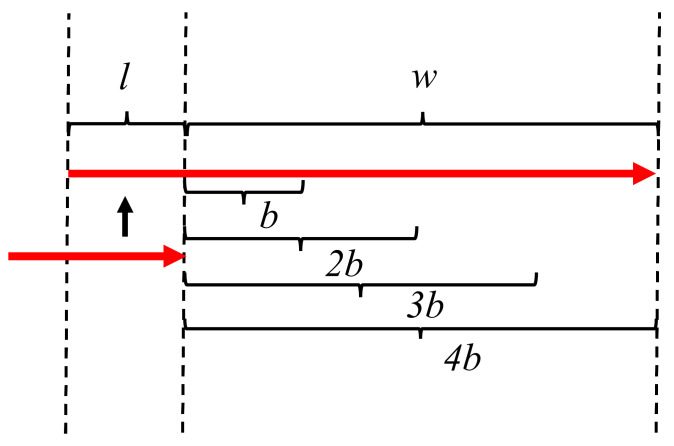
The red arrow represents a pose-graph window; the window size increases with every new measurement and is optimized after the vehicle travels every *b* meters [30].

The batch manager block incrementally optimizes the pose-graph for a given batch size *b* meters in the optimization window. Figure 7 provides an explanation of incremental pose-graph optimization, which is an extension of the work by Kümmerle et al. [12]. As the vehicle moves, edges received from the pose-graph reader are added to the optimizer. For every *b* meters traveled by the vehicle, the pose graph is optimized using one Gauss–Newton iteration. For a given window size, the number of optimization cycles decreases with an increase in batch size. The incremental optimization prevents the errors induced by the integration of vehicle odometry from growing out of proportion. It increases the optimizer’s stability while providing real-time optimized vehicle poses.

**Algorithm 2:** Incremental Hopping Window [30]

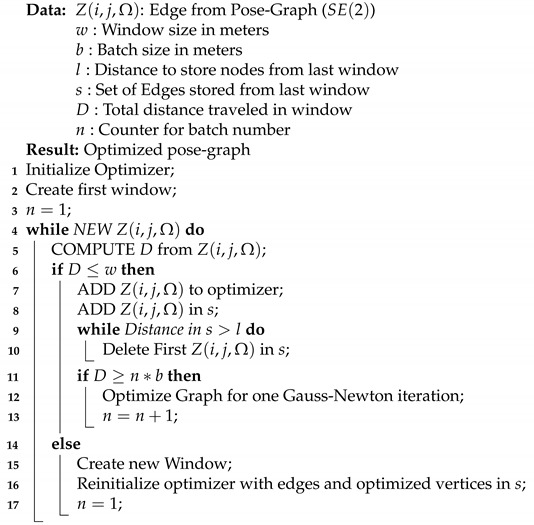



## 7. Experiments and Results

**Figure 8:**
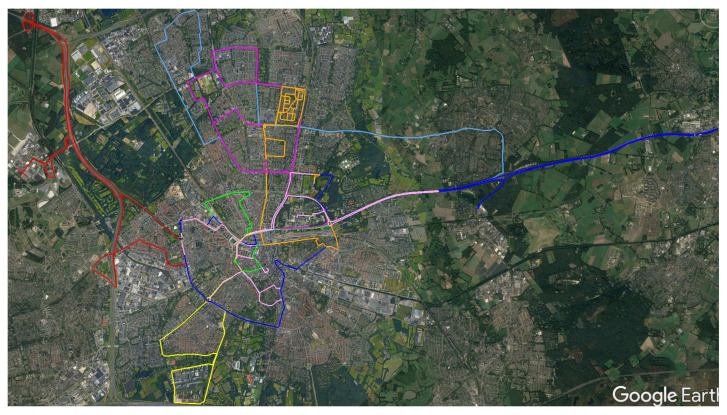
Overview of our eight datasets recorded around Eindhoven, The Netherlands. Each dataset is depicted in a different color [29].

We analyze the performance of the pose-graph based vehicle positioning and mapping framework on eight datasets covering more than 180 km of driving distance. The datasets are recorded with our test vehicle in driving sessions of 30 to 50 min in different environments like urban canyons, highways, and rural areas around the Eindhoven region in the Netherlands. The trajectories of all datasets are shown in Figure 8. We perform the following experiments for detailed analysis:

1. **GNSS data analysis:** Evaluation of the quality of GNSS readings from the receiver with respect to the RTK-GNSS. This sets the baseline for our other experiments.
2. **Graph modeling:** We compare and discuss the performance of the three graph modeling approaches provided in Section 4.
3. **Incremental hopping window performance analysis:** We analyze the performance of the incremental hopping window approach described in Section 6 in terms of positioning accuracy and robustness for different window sizes, batch sizes, and processing time.
4. **Performance analysis of the proposed framework:** We analyze the performance of the incremental hopping window approach using multiple odometry sources and a GNSS receiver.

### 7.1. Vehicle Setup

Our experimental vehicle is equipped with a stereo camera, a U-Blox GNSS receiver with PPP, an RTK-GNSS system, and a CAN interface to access the vehicle ECU messages. The stereo camera has a baseline of 30 cm composed of two PointGrey Firefly cameras. It captures images of 640 × 480 resolution at 60 Hz. The RTK-GNSS is the industry standard for accurate GNSS-based positioning and is postprocessed to obtain an accuracy of up to 0.01 m. The yaw-rate in rad/s and velocity in m/s is received at 25 Hz through the CAN interface from the vehicle ECU, and the U-Blox GNSS receiver operates at 1 Hz. The Pulse-per-second (PPS) from the GNSS receiver is used to synchronize the clock of the computer in the vehicle.

### 7.2. Performance Metrics

To evaluate the performance of our sensor fusion algorithm, we compare the results with the post-processed RTK-GNSS using the metrics described below. For both the GNSS readings and the fusion results, we compute these metrics for the poses corresponding to the PPS, which we call *PPS-Poses*.

1. **Maximum offset error** (Max.) in meters, which is the maximum offset Euclidean distance error over all *PPS-Poses* of each dataset computed with respect to the corresponding RTK-GNSS position. It gives an indication of the magnitude of the outliers in a dataset. It is computed as
  (3) $$Max.=\max_{1\leq i\leq n}\sqrt{{\left({\bar{X}}_{i}-{\hat{X}}_{i}\right)}^{2}+{\left({\bar{Y}}_{i}-{\hat{Y}}_{i}\right)}^{2}}\;,$$
  where *n* is the total number of poses in a dataset, $\bar{X}$, $\bar{Y}$ and $\hat{X}$, $\hat{Y}$ are the corresponding coordinate values of the positioning system GNSS or GNSS-Odometry fusion, and of the RTK-GNSS points respectively.
2. (Acc.) in meters, represents the structural offset between the RTK-GNSS and the positioning system under consideration. It is the Euclidean distance of the point computed from the average offset error in UTM-X and UTM-Y axes of the UTM coordinate system for each dataset. It is computed as
  (4) $$Acc.=\sqrt{\mu_{X}^{2}+\mu_{Y}^{2}}\;,$$
  where $\mu_{X}$ and $\mu_{Y}$ are the mean offset in the considered positioning system, computed as
  (5) $$\mu_{X}=\frac{1}{n}\sum_{i=1}^{n}{\bar{X}}_{i}-{\hat{X}}_{i}$$
  (6) $$\mu_{Y}=\frac{1}{n}\sum_{i=1}^{n}{\bar{Y}}_{i}-{\hat{Y}}_{i}.$$
3. **Precision** (Prec.) in meters, which is the standard deviation of the distance of each point from the computed mean offset error for UTM-X and UTM-Y axes for each dataset. It represents the variation or dispersion of the readings for the considered positioning system from its mean for each dataset. It is computed as
  (7) $$Prec.=\sqrt{\frac{1}{n-1}\sum_{i=1}^{n}D_{i}^{2}}\;,$$
  where $D_{i}$ is the distance of each point from the mean off-set, i.e.,
  (8) $$D_{i}=\sqrt{{\left({\bar{X}}_{i}-\mu_{X}\right)}^{2}+{\left({\bar{Y}}_{i}-\mu_{Y}\right)}^{2}}.$$

For these metrics, we also report the averages over all dataset and the relative percentage improvements of the pose-graph fusion with respect to the GNSS. As the relative information contained in the (visual) odometry cannot contribute to the absolute position information, we expect that our sensor fusion cannot improve accuracy (which measures the absolute position) but that it can improve on the maximum offset error and the precision.

### 7.3. Results

First, we analyze the GNSS receiver’s performance on the dataset against the RTK-GNSS receiver measurements in Section 7.3.1. It is considered the baseline for which the improvements in the performance metrics of the proposed framework is estimated. Secondly, we compare the performance of the three graph modeling approaches to our dataset and select the best performing in Section 7.3.2. Then, the incremental hopping window optimization performance on the selected pose-graph model is evaluated to estimate the correct batch size and optimization window size for real-time application in Section 7.3.3. Finally, in Section 7.3.4, we analyze the performance of the proposed framework with (i) vehicle odometry and GNSS fusion and (ii) SVO, vehicle odometry, and GNSS fusion, using the selected pose-graph model, the batch size, and the optimization window size.

#### 7.3.1. GNSS Data Analysis

**Figure 9:**
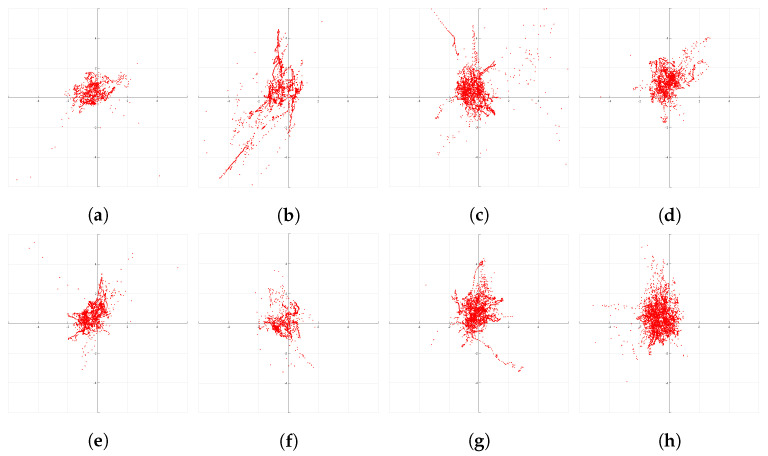
(**a**–**h**) shows the GNNS position errors with respect to the postprocessed RTK-GNSS ground truth for the eight datasets. The plots with a tile size of 2 × 2 m, clearly show that GNSS errors are biased and correlated, causing nonzero mean behavior for time spans up to 65 min [29].

First, we evaluate the quality of the GNSS data from the receiver with respect to the RTK-GNSS for all datasets. The performance metrics for the GNSS readings and the characteristic of the environment in which the majority of the datasets are recorded are shown in Table 1. The GNSS receiver performs well in a highway and rural environments. We can see that the precision for different datasets has an order of magnitude of a couple of meters, as expected. At the same time, we can see the maximum offset error varies a lot. This is because the GNSS signals suffered from reflection and occlusion in urban canyon areas and underpass. The *accuracy* of all the datasets is never close to zero due to a bias in the GNSS readings. This bias is clearly visible in the error’s scatter plots depicted in Figure 9. It is evident that over time spans that are relevant for automotive, that is, minutes to hours, the GNSS error distribution does not exhibit zero-mean behavior and that GNSS errors are highly correlated in time [29]. The existence of this bias is exactly the reason why RTK-GNSS base-stations need at-least 24 h or more averaging time to achieve a positioning accuracy of 2 cm. Fusing odometry measurements with GNSS readings can improve the *precision* of the positioning system.However, it cannot remove this bias, and therefore it cannot improve *accuracy*, as the vehicle odometry only provides information about the relative motions [42].

#### 7.3.2. Graph Modeling

The performance metrics of the modeling approaches *G1*, *G2*, and *G3* are shown in Table 2. For the first graph modeling approach *G1*, the average maximum offset error decreased by 21.84%, but the average precision increased by 6.75%, which is unwanted. The average accuracy remained the same as the average GNSS accuracy, as expected. It is observed that out of the eight experiments, only three converged for the approach *G1*. We believe this convergence issue is caused by too rigid modeling of the pose-graph, which hampers convergence when the vehicle poses, initialized from the odometry, are far off from the GNSS readings.

The pose-graph modeling approach *G2* performed much better than *G1*. The optimization converged for all eight experiments. Table 2 shows the performance metrics for this approach. The average maximum offset error and precision decreased by 69.53% and 17.79%, respectively. It performed well in both highways and urban-area scenarios. The improvement with respect to the first model *G1* is due to the fact that model *G2* has more flexibility, as the GNSS positions are also optimized. It improves convergence for scenarios with a challenging initialization when there is a large deviation of the odometry heading to the GNSS heading.

The pose-graph modeling approach *G3* performed well, but the performance was less than *G2*. Table 2 shows the performance metrics of this approach. The average maximum offset error and precision decreased by 49.47% and 15.34%, respectively. Like *G1* and *G2*, there is no significant change in the average accuracy, as expected.

#### 7.3.3. Incremental Hopping Window Analysis

In the previous experiments, we have optimized the entire pose-graph offline, where the modeling approach *G2* performed best. In this section, we study the performance achieved when optimizing the pose-graph structure *G2* online with the incremental hopping window optimization strategy. It provides valuable insight into the reliability and real-time applicability of the localization system. We also study the impact of different window sizes *w* and batch sizes *b* on the performance metrics. The incremental hopping window optimization strategy is evaluated on eight datasets with five different window sizes (500, 1000, 1500, 2000, and 2500 m). For each window size, we perform optimization for 16 different batch sizes (5, 10, 20, 25, 40, 50, 66, 100, 111, 125, 142, 166, 200, 250, 333, and 500 m). The proposed strategy runs on a single CPU core of a multicore Intel CPU running at 2.3 GHz. Table 3 shows the average of the performance metrics of all datasets for a window size of 1000 m and a batch size of 5 m. As expected, both precision and the maximum error are improved significantly with respect to GNSS, but the accuracy is not improved due to the bias in GNSS errors.

**Figure 10:**
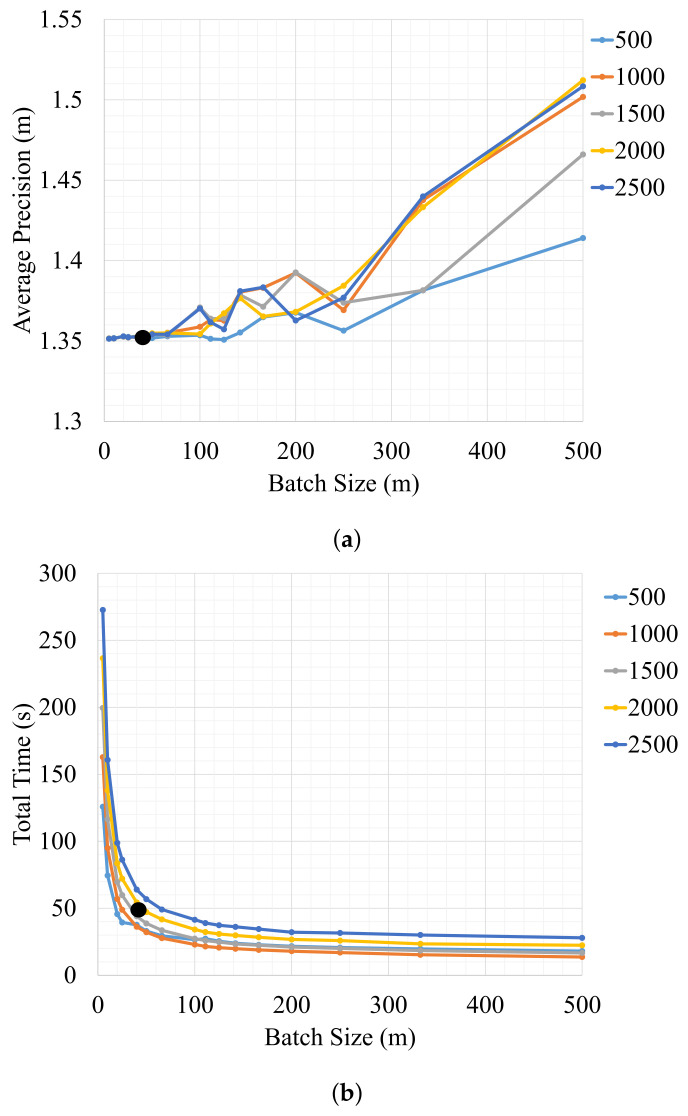
(**a**) Batch size vs. average precision plot for different window sizes (500, 1000, 1500, 2000, 2500 in meters) of all datasets [30]. (**b**) Batch size vs. total time plot for different window sizes. The configuration with a window of 1500 m and a batch size of 40 m is marked with a black dot [30].

Figure 10a shows the influence of the batch size on the average precision over all datasets. The incremental hopping window strategy provides the best results when optimizing with a batch size of 5 m for different window sizes. It performs in total 200 Gauss-Newton iterations for each window of size 1000 m. The precision is similar for batch sizes between 5 and 66 m but degrades for larger batch sizes. This shows that the precision of the system is highly dependent on the batch size rather than the window size. It is observed that the difference in precision between incremental hopping window optimization and global optimization is only 1 centimeter, which is in range of the accuracy of the ground truth. Therefore, in our experiments, both global optimization using all measurements and local optimization using the proposed framework achieve similar accuracy, as desired. Figure 10b shows the batch size versus total computation time considering all datasets. The computation time increases with decreasing batch sizes, and it follows a similar pattern for different window sizes. This is because the total number of Gauss–Newton iterations increases with decreasing batch size.

**Figure 11:**
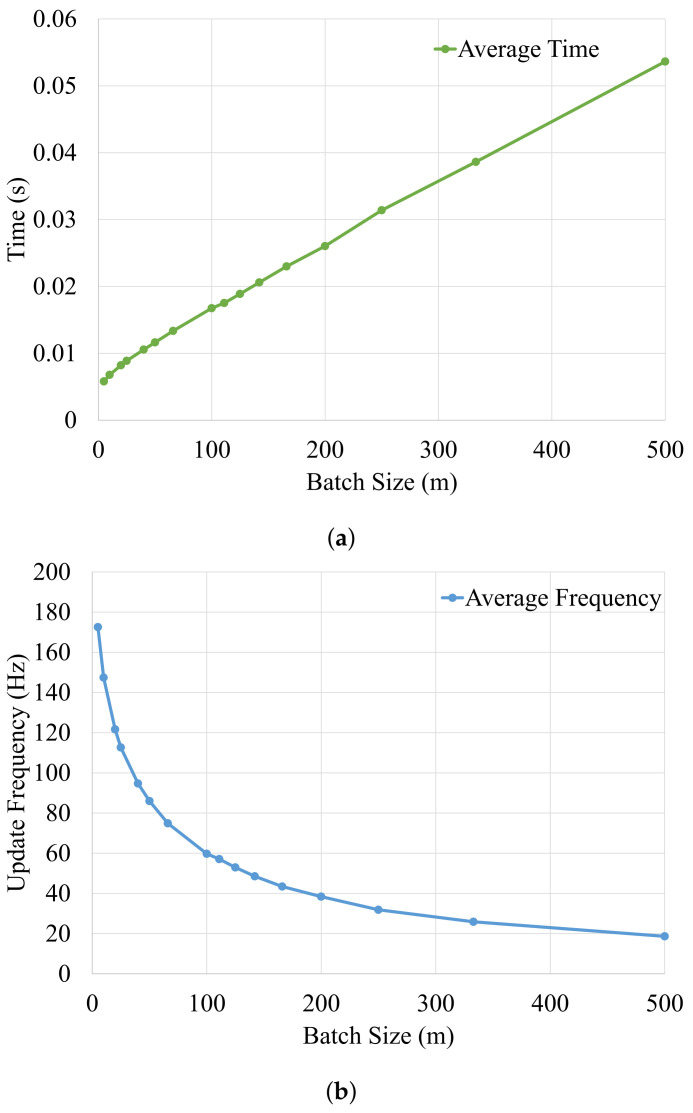
(**a**,**b**) shows the average computation time and the update frequency for one optimization iteration for different batch sizes, respectively [30].

Figure 11 provides the computation times and update rates for different batch sizes to demonstrate the real-time capability of the hopping-window optimization strategy. Here we can see that the computation time increases linearly with increase in batch size. Keeping in mind that the vehicle odometry is provided at 25 Hertz, it can be seen that practically all batch sizes meet this real-time constraint of 25 Hertz. An optimization window of 1500 m and a batch size of 40 m provide a good trade-off between precision and computation time. This configuration is marked with the black dot in Figure 10.

#### 7.3.4. Performance Analysis of the Proposed Framework

The previous experiments show that the graph model *G2* performs best in our large dataset. The Incremental hopping window pose-graph optimization with a batch size of 40 m and a window size of 1500 m provides a balanced configuration for real-time application. We choose these settings to analyze the performance of the proposed positioning and mapping framework with (i) vehicle odometry and GNSS fusion and (ii) SVO, vehicle odometry, and GNSS fusion. The performance metrics of the proposed framework are shown in Table 4. GO1 represents offline global optimization, and LO1 represents incremental hopping window optimization both using vehicle odometry and GNSS data as input. GO2 represents offline global optimization, and LO2 represents incremental hopping window optimization both using SVO, vehicle odometry, and GNSS data as input. We can see that GO1 and LO1 show significant improvement over GNSS data, with GO1 performing marginally better than LO1. GO1 shows a 69.53% improvement in maximum offset error and 17.79% improvement in the error’s standard deviation when compared with automotive-grade GNSS receivers. In comparison, LO1 shows a 60.52% improvement in maximum offset error and 17.18% improvement in the error’s standard deviation. It shows that the incremental hopping window optimization with a batch size of 40 m and a window size of 1500 m performs similarly with respect to global optimization of the pose-graph.

**Figure 12:**
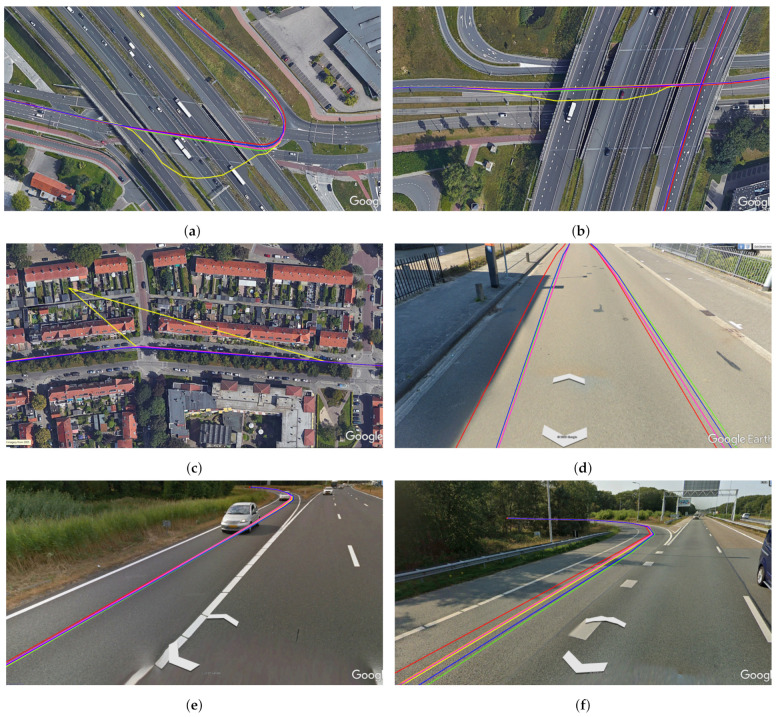
(**a**–**c**) shows the fusion results projected onto the Google map. (**d**–**f**) shows the fusion results projected on to the Google street view. Where, RTK GNSS (red), GNSS (yellow), Offline Global optimization of vehicle odometry and GNSS (GO1) in blue, incremental hopping window optimization of vehicle odometry and GNSS (LO1) in green, Offline Global optimization of SVO, vehicle odometry and GNSS (GO2) in orange, incremental hopping window optimization of SVO, vehicle odometry and GNSS (LO2) in pink.

Then we perform experiments to fuse SVO, vehicle odometry, and GNSS data. We see similar trends to the last experiments where both GO2 and LO2 showed significant improvement over GNSS data, with GO2 performing marginally better than LO2. GO2 shows a 65.49% improvement in maximum offset error and 20.86% improvement in the error’s standard deviation when compared with automotive-grade GNSS receivers. Whereas LO2 shows a 65.45% improvement in maximum offset error and 20.25% improvement in the error’s standard deviation. We also observe that GO2 performed better than GO1 and LO2 performed better than LO1, which shows that SVO improves the performance of the positioning system. It also shows that the SVO is more accurate than vehicle odometry, but vehicle odometry is more robust than SVO. Figure 12 shows some pictures of the results of all of the fusion approaches projected onto Google maps. Figure 12a,b show that the GNSS reading represented as a yellow line degraded while the vehicle was traveling under a bridge. However, all of the fusion approaches handled the situation well and remained close to the RTK-GNSS reading represented with a red line. Figure 12c shows a similar situation when the GNSS readings degraded when the vehicle was traveling under trees, and the fusion algorithms remained close to the RTK-GNSS readings. Figure 12d–f show the results of the fusion approaches projected onto the Google street view.

## 8. Conclusions

We have proposed and evaluated a pose-graph based real-time multi-sensor fusion framework for vehicle positioning and mapping using a stereo camera, vehicle’s yaw-rate, velocity sensor, and a GNSS receiver. The framework is extensively evaluated on a dataset with 180 km of vehicle trajectories recorded in highway, urban, and rural areas. It is shown that the graph model in which the GNSS readings are modeled as optimizable nodes (approach *G3*), achieves the best results in our experiments, as it allows for more flexibility and thereby improves convergences. The precision of incremental hopping window optimization is shown to be very similar to that of global optimization. The difference is only 1%, which indicates that the most valuable information for sensor fusion for vehicle positioning is contained in sensor readings of the last 500 m of the vehicle trajectory. It is shown that performing frequent iterations for incremental hopping window strategy by using a small batch size improves the precision with an increase in the optimization time. A window size of 1500 m and a batch size of 40 meters produced a balanced result, in terms of computation time and positioning precision. We have analyzed the framework’s performance with (i) vehicle odometry and GNSS fusion, and (ii) stereo visual odometry, vehicle odometry, and GNSS fusion. The results show that the pose-graph approach, which models stereo visual odometry (SVO), vehicle odometry, and GNSS data, and which is optimized offline (approach GO2), performs overall best in our dataset. It showed a 20.86% improvement in precision when compared to a GNSS receiver, whereas the accuracy remains the same. This is expected as the accuracy of the positioning system is bounded by the accuracy of the GNSS receiver. We can conclude that the stereo visual odometry improves the precision of the positioning system when compared to vehicle odometry and GNSS fusion, and the incremental hopping window pose-graph optimization (approach LO1 and LO2) performs similar to offline global optimization (approach GO1 and GO2) in our dataset.

## Figures and Tables

**Table 1:** Performance metrics of GNSS receiver.

Dataset	*Max.*	*Acc.*	*Prec.*	Environment
1	66.29	0.489	2.65	Highway, Underpass
2	21.80	0.87	2.49	Urban canyon, Rural
3	49.07	0.72	2.29	Highway, Underpass
4	4.83	1.09	1.03	Rural, Urban
5	16.78	0.68	1.14	Urban canyon, Rural
6	3.67	0.34	0.95	Highway, Rural
7	7.76	0.78	1.18	Urban, Highway
8	18.04	0.81	1.26	Urban
**Average**	**23.53**	**0.72**	**1.63**	

**Table 2:** Performance metrics table for Graph model *G1*, *G2*, and *G3*.

Dataset	Maximum Offset Error			Accuracy			Precision		
	***G1***	***G2***	***G3***	***G1***	***G2***	***G3***	***G1***	***G2***	***G3***
1	9.57	6.28	10.62	0.45	0.46	0.44	1.03	0.96	1.04
2	20.84	14.17	20.08	0.88	0.79	0.86	2.55	2.34	2.38
3	28.51	14.02	27.15	0.71	0.73	0.71	2.01	1.68	1.98
4	16.24	4.85	5.41	1.14	1.09	1.10	1.69	1.13	1.05
5	32.26	4.27	6.84	0.70	0.65	0.66	2.13	1.01	0.99
6	15.50	3.12	3.05	0.31	0.32	0.34	1.17	0.93	0.93
7	6.17	5.30	15.19	0.78	0.80	0.76	1.22	1.30	1.36
8	18.05	5.37	6.76	0.82	0.80	0.80	2.15	1.35	1.28
**Average**	**18.39**	**7.17**	**11.89**	**0.72**	**0.71**	**0.71**	**1.74**	**1.34**	**1.38**
**Improvement w.r.t. GNSS (%)**	**21.84**	**69.53**	**49.47**	**0.00**	**1.39**	**1.39**	**−6.75**	**17.79**	**15.34**

**Table 3:** Performance metric table for Window size 1000 m and Batch size 5 m.

Dataset	*Max.*	*Acc.*	*Prec.*
1	6.68	0.44	0.99
2	18.61	0.85	2.37
3	24.36	0.71	1.91
4	5.04	1.10	1.07
5	4.23	0.65	1.03
6	2.93	0.33	0.94
7	4.96	0.79	1.23
8	7.49	0.81	1.28
**Average**	**9.29**	**0.71**	**1.35**
**Improvement w.r.t. GNSS %**	**60.53**	**1.43**	**16.81**

**Table 4:** Performance metrics table for different pose-graph fusion approach: Offline global optimization with vehicle odometry, and GNSS data (GO1), incremental hopping window optimization with vehicle odometry and GNSS data (LO1), Offline global optimization with stereo visual odometry, vehicle odometry and GNSS data (GO2), incremental hopping window optimization with stereo visual odometry, vehicle odometry and GNSS data (LO2).

Dataset	Maximum Offset Error				Accuracy				Precision			
	**GO1**	**LO1**	**GO2**	**LO2**	**GO1**	**LO1**	**GO2**	**LO2**	**GO1**	**LO1**	**GO2**	**LO2**
1	6.28	6.68	5.99	5.99	0.46	0.45	0.45	0.45	0.96	1.00	0.93	0.93
2	14.17	18.61	16.61	16.54	0.79	0.85	0.84	0.85	2.34	2.37	2.31	2.32
3	14.01	24.36	17.87	17.99	0.73	0.71	0.67	0.67	1.68	1.91	1.73	1.76
4	4.85	5.04	4.72	4.73	1.09	1.10	1.09	1.09	1.13	1.07	1.06	1.05
5	4.27	4.23	3.99	4.00	0.65	0.65	0.65	0.65	1.01	1.03	0.97	0.96
6	3.12	2.93	2.94	2.96	0.32	0.33	0.33	0.33	0.93	0.94	0.95	0.95
7	5.30	4.96	4.53	4.52	0.80	0.79	0.77	0.77	1.29	1.23	1.17	1.17
8	5.37	7.49	8.29	8.28	0.80	0.81	0.81	0.80	1.35	1.28	1.24	1.24
**Average**	**7.17**	**9.29**	**8.12**	**8.13**	**0.71**	**0.71**	**0.70**	**0.70**	**1.34**	**1.35**	**1.29**	**1.30**
**Improvement w.r.t. GNSS %**	**69.53**	**60.52**	**65.49**	**65.45**	**1.39**	**1.39**	**2.78**	**2.78**	**17.79**	**17.18**	**20.86**	**20.25**

## Data Availability

Not applicable.
